# *Hericium erinaceus* Protein Alleviates High-Fat Diet-Induced Hepatic Lipid Accumulation and Oxidative Stress In Vivo

**DOI:** 10.3390/foods14030459

**Published:** 2025-01-31

**Authors:** Hongzheng Lu, Siqi Yang, Wei Li, Baodong Zheng, Shaoxiao Zeng, Haoran Chen

**Affiliations:** 1 Engineering Research Centre of Fujian-Taiwan Special Marine Food Processing and Nutrition (Ministry of Education), Fujian Agriculture and Forestry University, Fuzhou 350002, China; 2 College of Food Science, Fujian Agriculture and Forestry University, Fuzhou 350002, China

**Keywords:** *Hericium erinaceus* protein, pancreatic lipase, hepatic steatosis, PPARα

## Abstract

Dietary interventions with food-derived natural products have emerged as a promising strategy to alleviate obesity. This study aims to investigate the anti-obesity effect of *Hericium erinaceus* protein (HEP) and its underlying mechanism. Our results demonstrated that HEP exhibited excellent radical scavenging activity in vitro. In vivo, HEP intervention reduced pancreatic lipase activity in the intestine and enhanced fat excretion, thereby inhibiting the absorption of dietary fats. Meanwhile, HEP ameliorated the body weight and organ indexes, dyslipidemia, insulin resistance, hepatic steatosis, and liver oxidative stress injuries in obese mice. The results of real-time PCR (qRT-PCR) and Western blot analyses indicated that HEP upregulated the expression of peroxisome proliferator-activated receptor α (PPARα), subsequently upregulated the expression of liver fatty acid oxidation-related genes (lipoprotein lipase (LPL), carnitine palmitoyltransferase 1a (CPT-1a), and acyl-CoA oxidase 1 (ACOX1)) and downregulated the expression of lipogenesis-related genes (sterol regulatory element-binding protein-1c (SREBP-1c), stearoyl-coenzyme A desaturase 1 (SCD-1), and fatty acid synthase (FASN)), thereby ameliorating lipid metabolism disorders. Therefore, these findings demonstrated that HEP exerted protective effects on lipid metabolism disorders by activating the PPARα pathway, indicating its potential as a dietary supplement for the prevention and amelioration of obesity.

## 1. Introduction

Obesity is a major global public health challenge in the 21st century, with its prevalence steadily increasing. According to the World Health Organization, the global prevalence of overweight and obesity has been rising since the 1970s, and by 2022, the number of obese individuals worldwide exceeded one billion, representing approximately one-eighth of the global population [1]. This trend poses a threat to individual health and places a heavy burden on the global economy, with obesity-related diseases causing direct and indirect economic losses estimated at USD 2 trillion annually [2]. Notably, the obesity epidemic is closely linked to modern lifestyle changes and dietary habits, with an imbalance between excessive energy intake and reduced physical activity being the main cause [3]. Long-term obesity leads to fat ectopic accumulation in the body, which induces type II diabetes, non-alcoholic fatty liver disease, hypertension, and other metabolic disorders [4,5].

Despite advances in therapeutic strategies for obesity, existing pharmacologic treatments have significant limitations. Many weight-loss drugs, while effective in managing body weight, are accompanied by side effects such as diarrhea and flatulence. Furthermore, concerns regarding drug dependence limit their safety and feasibility for long-term use [6]. Recently, numerous studies have demonstrated that food-derived natural products (e.g., proteins, polysaccharides, polyphenols, terpenoids, etc.) exhibit hypolipidemic, hypoglycemic, and antioxidant properties [7]. In particular, plant-derived natural products, which are abundant, demonstrate higher bioactivity and lower toxicity, making them highly promising for the development of anti-obesity drugs [8]. Therefore, screening plant-derived natural products for anti-obesity properties represents a promising direction for future research.

*Hericium erinaceus*, also known as Yamabushitake or Lion’s mane mushroom, is a valuable medicinal fungus that primarily grows on oaks or holm-oaks. *Hericium erinaceus* is rich in nutrients and contains various biologically active substances, including proteins, polysaccharides, oligosaccharides, vitamins, cellulose, and baobabin [9]. *Hericium erinaceus* has been demonstrated to possess numerous health-promoting properties, such as hypolipidemic, anti-inflammatory, antioxidant, and anti-diabetic functions [10,11]. Consequently, *Hericium erinaceus* extracts are gaining increasing attention for their health benefits, particularly their efficacy in improving glucose and lipid metabolism disorders. It was reported that oral administration of dietary fiber from *Hericium erinaceus* significantly reduced HFD-induced oxidative stress and liver injury, thereby exerting hypolipidemic effects [12]. Choi et al. found that ethanolic extracts from *Hericium erinaceum* could improve HFD-induced dyslipidemia in rats [13]. *Hericium erinaceus* insoluble dietary fiber increased the abundance of *Akkermansia* and *norank_f_Muribaculaceae* in the gut microbiota of obese mice, thereby ameliorating obesity [14]. Similarly, Cai et al. demonstrated that *Hericium erinaceus* polysaccharides increased antioxidant enzyme activity in STZ-induced type II diabetic rats and attenuated hepatic damage and dyslipidemia, thereby lowering fasting blood glucose levels [15]. However, studies on the physiological efficacy of *Hericium erinaceus* protein (HEP) are still at an early stage compared with polysaccharides and dietary fibers. *Hericium erinaceus* has been reported to contain about 25% protein with a balanced amino acid composition [16]. Previous studies indicated that HEP exhibited potential anti-tumor activity by remodeling the gut microbiota composition in mice and reducing inflammatory response [17]. Chen et al. demonstrated that HEP has an immunomodulatory effect on the gut microbiota, suggesting its use as an immunotherapeutic drug or functional food ingredient [18]. These findings highlight the health benefits of HEP, particularly its potential in regulating gut microbiota homeostasis, enhancing immunity, and reducing inflammation. However, there is still a gap on how HEP ameliorates lipid metabolism disorders, with an urgent need for further studies to elucidate the mechanism by which HEP can prevent and ameliorate obesity.

Therefore, the aim of this study was to explore the ameliorative effect and mechanism of HEP in obese mice. Firstly, the compositional properties and antioxidant capacity of HEP in vitro were evaluated. Next, an obesity model was established using HFD feeding C57BL/6J mice, and improvement by HEP on body weight, organ index, blood lipid and glucose levels, hepatic oxidative stress, and histopathology were determined. Finally, the expression of hepatic lipid metabolism-related genes and proteins was analyzed using qRT-PCR and Western blot to investigate the mechanism of HEP to ameliorate obesity.

## 2. Methods

### 2.1. Materials and Reagents

*Hericium erinaceus* was purchased from the Walmart supermarket (Fuzhou, China). High-density lipoprotein cholesterol (HDL-C), low-density lipoprotein cholesterol (LDL-C), total cholesterol (TC), triglycerides (TG), glucose, alanine aminotransferase (ALT), and aspartate aminotransferase (AST) kits were purchased from Jiancheng (Nanjing, China). Superoxide dismutase (SOD), catalase (CAT), glutathione peroxidase (GSH-Px), and Malondialdehyde (MDA) kits were obtained from Beyotime (Shanghai, China). Ultrapure RNA extraction kits and Cham Q SYBR qPCR Master Mix were obtained from TransGen (Beijing, China). The BCA assay kit and RIPA lysis buffer were obtained from Solarbio (Beijing, China). Primary antibodies PPARα, CPT-1a, ACOX1, SREBP-1c, SCD-1, and FASN were purchased from Proteintech (Wuhan, China).

### 2.2. Preparation of HEP

*Hericium erinaceus* was pulverized using an ultra-micro pulverizer and sieved through a 100-mesh sieve. A total of 5 g of *Hericium erinaceus* powder was mixed with 100 mL of pure water. The pH was adjusted to 10 and stirred at 40 °C for 4 h. Then, the mixture was centrifuged at 8000× *g* for 10 min, the supernatant was collected, the pH was adjusted to 4.5, and the mixture was centrifuged at 8000× *g* for 10 min. The precipitate was collected, and the pH was adjusted to neutral and dialyzed with deionized water (3000 Da) for 24 h. Finally, the HEP was obtained by vacuum freeze-drying.

### 2.3. Composition of HEP

The protein content of HEP samples was measured by a Kjeldahl nitrogen meter. The carbohydrate content was measured using the method outlined by Hu et al. [19]. The moisture content was assessed by drying the HEP samples at 105 °C for 12 h to calculate the weight loss. Additionally, the ash content was measured by combustion at 550 °C.

### 2.4. In Vitro Antioxidant

HEP samples were solubilized in distilled water (0.1, 0.25, 0.5, 1, and 2 mg/mL). Then, the Fe^2+^ chelating capacity, ABTS radical scavenging capacity, hydroxyl radical scavenging capacity, and DPPH radical scavenging capacity of HEP were assayed following a previous method [20,21]. Vitamin C (VC) was a positive control.

### 2.5. Animals

**Figure 1:**
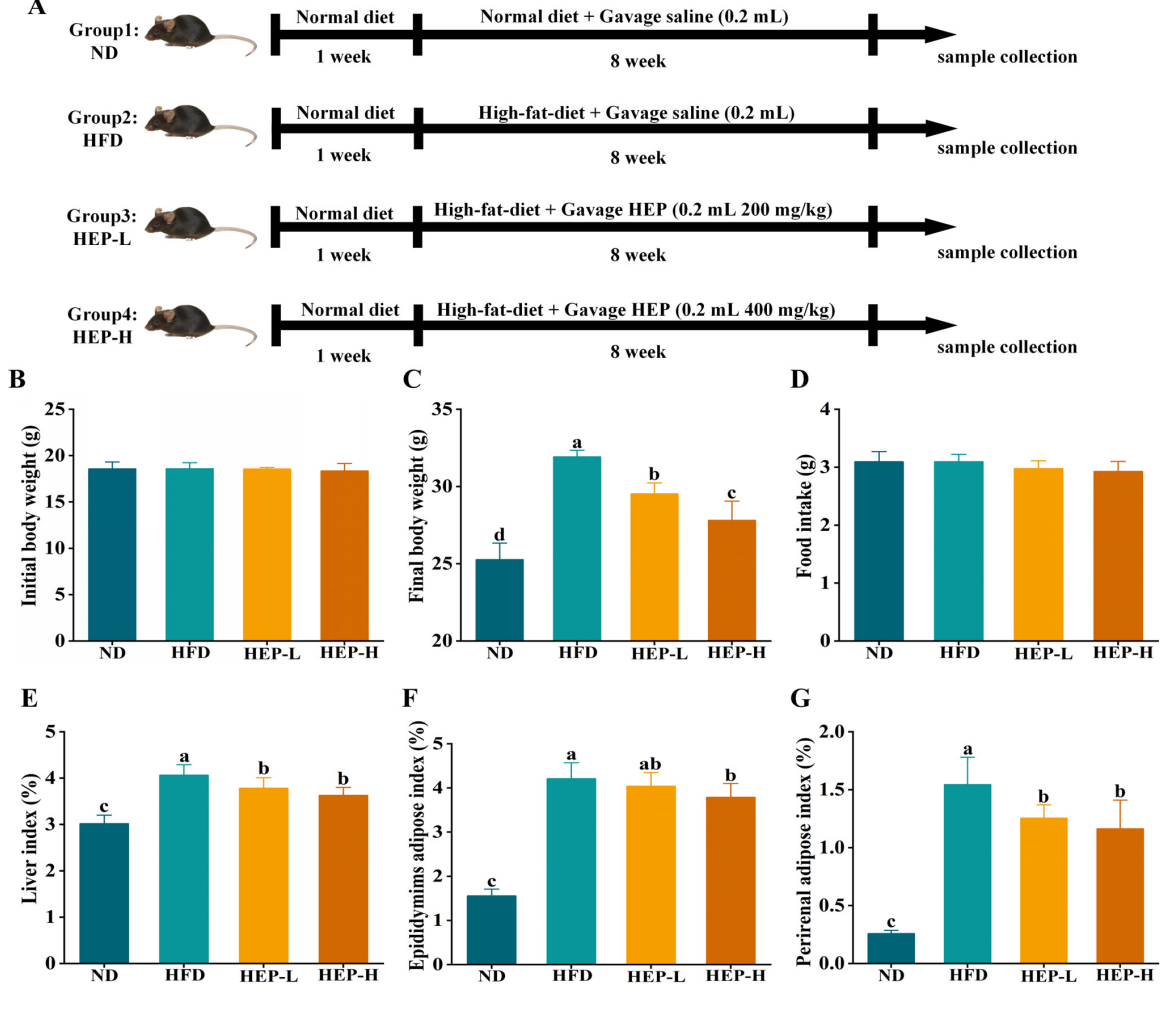
Animal experimental design and effect of HEP on physiological indicators in obese mice: (**A**) Animal experimental design of the present study; (**B**) initial body weight; (**C**) final body weight; (**D**) food intake; (**E**) liver index; (**F**) epididymal adipose index; (**G**) perirenal adipose index. The different lowercase letters (a, b, c, d) indicate significant differences between values in different groups (*p* < 0.05).

Four-week-old male C57BL/6J mice were kept on a 12 h light/dark cycle at a temperature of 25 ± 2 °C and humidity of 50 ± 5%. Water and food were provided freely. After seven days of acclimatization feeding, the mice were randomly divided into four groups (6 mice per group) (Figure 1A) as follows: Normal group (ND): mice were fed a normal fat diet (10% of energy coming from fat) with 0.2 mL of saline by gavage once a day; high-fat diet group (HFD): mice were fed a high-fat diet (60% of energy coming from fat) with 0.2 mL of saline by gavage once a day; low-dose HEP intervention: mice were fed HFD with 200 mg/kg.bw HEP by gavage once a day; high-dose HEP intervention: mice were fed HFD with 400 mg/kg.bw HEP by gavage once a day. The dosage of HEP administered was based on a previous study [22,23]. The composition of the diets is listed in Table S1. At the end of 8 weeks, blood samples were collected, and the mice were euthanized for organ removal. Organ index = organ weight/body weight × 100%. All of the animal procedures were approved by the Animal Care and Use Committee of Fujian Agriculture and Forestry University (Ethical approval number: PZCASFAFU23076).

### 2.6. Fecal TG Levels

The fecal TG was obtained by a previous method [24]. Then, the TG content was determined using a biochemical analysis kit. The protein content was determined by the BCA assay kit.

### 2.7. Pancreatic Lipase Activity in the Small Intestine

In short, the luminal contents of the small intestine were mixed with PBS and then centrifuged at 10,000× *g* for 10 min to collect the supernatant. The pancreatic lipase activity was determined using an ELISA kit (Meiman, China).

### 2.8. Serum Biochemical Analysis

Serum TG, TC, LDL-C, HDL-C, ALT, AST, and glucose levels were measured using biochemical kits. Insulin levels were determined using an ELISA kit (Meiman, China). The insulin resistance index (HOMA-IR) and insulin sensitivity index (HOMA-IS) were calculated based on serum glucose and insulin levels.

### 2.9. OGTT

In the 7th week, mice were fasted overnight and then gavaged with glucose (2 g/kg body weight). Blood glucose levels were measured by a glucometer at 0, 30, 60, 90, and 120 min. The area under the curve (AUC) of the glucose levels was calculated.

### 2.10. Liver TG and TC Content

The lipids were extracted from the liver (100 mg) homogenate using Folch’s method. Then, the liver TG and TC levels were assayed by biochemical kits. Meanwhile, the protein content was quantified by a BCA assay kit.

### 2.11. Liver Injury Analysis

A total of 100 mg of liver tissue was homogenized in 1 mL of PBS and centrifuged at 8000× *g* for 15 min, and the supernatant was collected. Liver SOD, GSH-Px, CAT, and MDA levels were assayed by biochemical kits. Meanwhile, the protein content was quantified by a BCA assay kit.

### 2.12. H&E Staining

Liver and epididymal adipose were fixed in 10% formalin buffer for 24 h. Then, paraffin sections were stained with H&E. The images of paraffin sections were observed and photographed under a microscope and were quantified using Image J V1.8.0.112.

### 2.13. qRT-PCR

Total RNA was extracted from the liver using an ultrapure RNA extraction kit. cDNA was synthesized from the RNA using a reverse transcription kit. Then, quantitative RT-PCR was performed using the SYBR qPCR Master Mix with a Real-Time PCR system. β-actin, as the reference gene, and the relative mRNA expression of the target genes were calculated using the 2-ΔΔCT method. The primer sequences are shown in Table S2.

### 2.14. Western Blot

The liver was lysed in RIPA buffer containing protease and phosphatase inhibitors. Protein samples (50 μg) were separated by SDS-PAGE and transferred to a nitrocellulose membrane. The membrane was blocked with 5% bovine serum albumin for 1 h, then incubated with primary antibody at 4 °C overnight. The membrane was incubated with a secondary antibody (Proteintech, China) for 1 h. Finally, ECL Plus ultrasensitive luminescent solution was applied to the membrane, which was then imaged using a gel imager and quantified with Image J.

### 2.15. Statistical Analysis

Data were expressed as means ± SD. Differences between groups were analyzed by one-way analysis of variance (ANOVA) followed by Duncan’s multiple comparison test. *p* < 0.05 indicated statistical significance. All the data were analyzed using SPSS 20.0.

## 3. Results

### 3.1. The Compositional Properties of HEP

The composition of HEP powder is listed in Table S3. The HEP powder contains 86.57% protein, 4.67% carbohydrates, 6.03% moisture, and 2.28% ash, indicating that HEP powder has a high protein purity.

### 3.2. In Vitro Antioxidant Capacity of HEP

**Figure 2:**
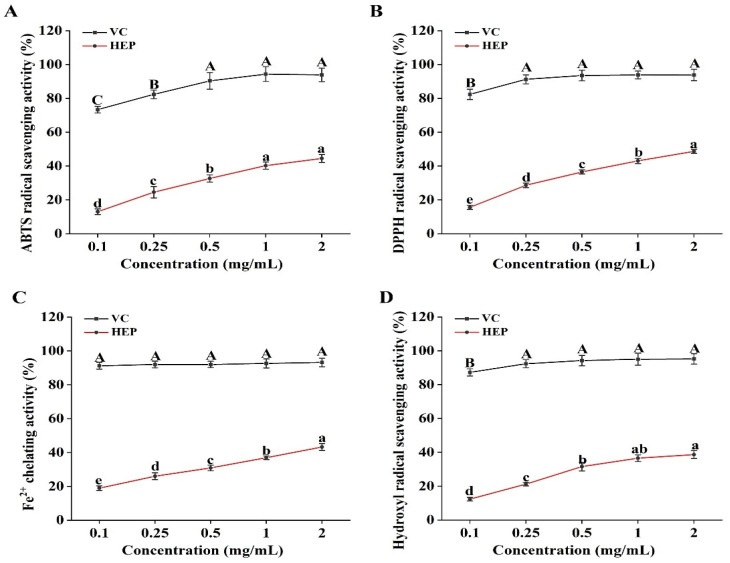
The antioxidant activities of HEP in vitro: (**A**) ABTS radical scavenging activity; (**B**) DPPH radical scavenging activity; (**C**) Fe^2+^ chelating activity; (**D**) hydroxyl radical scavenging activity. The different uppercase letters (A, B, C) indicate significant differences between values in different VC groups (*p* < 0.05). The different lowercase letters (a, b, c, d, e) indicate significant differences between values in different HEP groups (*p* < 0.05).

As shown in Figure 2A,B, the ABTS and DPPH radical scavenging activities of HEP increased gradually with concentration, reaching 44.52% and 48.69% at 2.0 mg/mL, respectively (*p* < 0.05). Similarly, HEP exhibited a significant dose-dependent effect on Fe^2+^ chelating activity and hydroxyl radical scavenging activity, achieving 43.25% and 38.69% at 2.0 mg/mL, respectively (Figure 2C,D, *p* < 0.05). However, despite exhibiting some antioxidant activity, HEP was significantly weaker compared with VC (Figure 2A–D, *p* < 0.05).

### 3.3. Effect of HEP on Physiological Indicators in Obese Mice

As outlined in Figure 1B, there was no significant difference in the initial weight of the mice among all groups. However, after 8 weeks of HFD feeding, the body weight in the HFD group was enhanced by 1.26-fold in comparison with the ND group (Figure 1C, *p* < 0.05). Encouragingly, the HEP intervention significantly reduced the body weight in a dose-dependent manner (*p* < 0.05). As illustrated in Figure 1D, there was no significant difference in food intake among all groups, indicating that the HEP intervention did not alter the appetite (*p* > 0.05). Additionally, compared to the ND group, the liver index, epididymal adipose index, and perirenal adipose index in the HFD group were increased (Figure 1E–G, *p* < 0.05). In comparison with the HFD group, the HEP intervention remarkably reversed these trends (Figure 1E–G, *p* < 0.05). However, the body weight and organ index were significantly higher in the HEP-treated group than in the ND group (Figure 1, *p* < 0.05). Thus, our results indicated that HEP could significantly alleviate body weight and organ index gain in obese mice without altering the appetite.

### 3.4. Effect of HEP on Dietary Fat Absorption in Obese Mice

**Figure 3:**
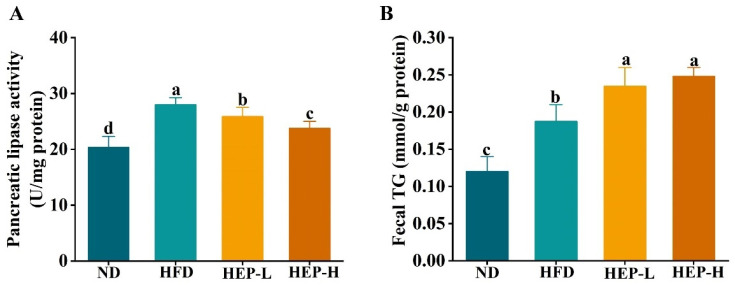
Effects of HEP on pancreatic lipase activity and fecal TG levels in obese mice: (**A**) Pancreatic lipase activity and (**B**) fecal TG. The different lowercase letters (a, b, c, d) indicate significant differences between values in different groups (*p* < 0.05).

As shown in Figure 3A, the activity of pancreatic lipase in the HFD group was remarkably enhanced compared with the ND group, indicating that fat hydrolysis efficiency was accelerated in obese mice (*p* < 0.05). The HEP intervention dose-dependently inhibited pancreatic lipase activity (Figure 3A, *p* < 0.05). Notably, the pancreatic lipase activity in the HEP-treated group did not return to normal levels (Figure 3A, *p* < 0.05). Meanwhile, we also measured the fecal TG content of mice. The fecal TG levels in the HFD group were remarkably increased (Figure 3B, *p* < 0.05). Interestingly, the fecal TG levels in the HEP-L and HEP-H groups were higher than those in the HFD group, which may be attributed to the inhibition of pancreatic lipase activity by HEP (Figure 3B, *p* < 0.05).

### 3.5. Effect of HEP on Serum Lipid Levels in Obese Mice

**Figure 4:**
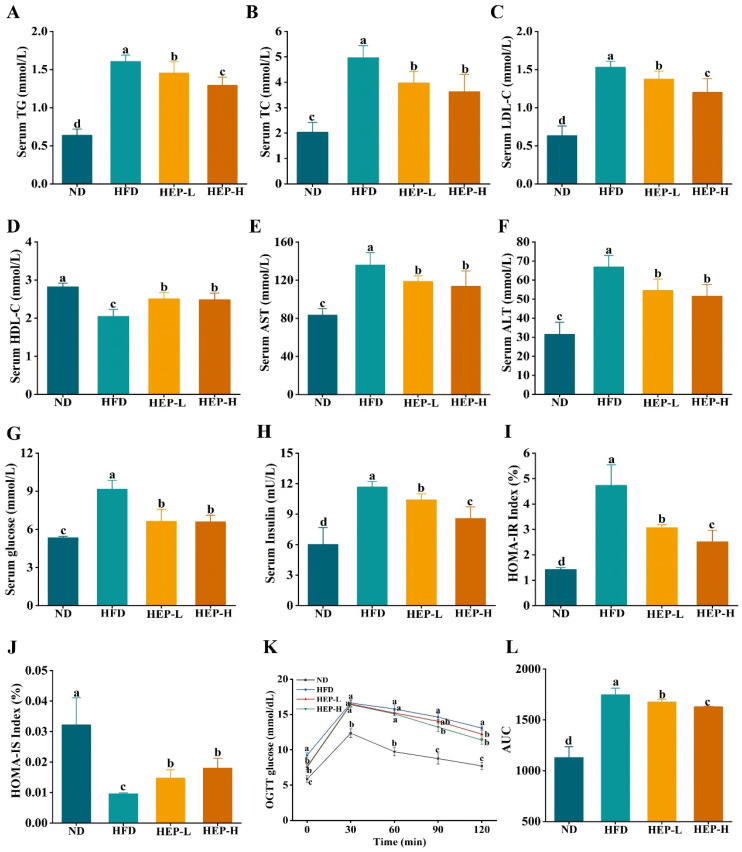
Effect of HEP on serum biochemical indicators in obese mice: (**A**) Serum TG levels; (**B**) serum TC levels; (**C**) serum LDL-C levels; (**D**) serum HDL-C levels; (**E**) serum AST activities; (**F**) serum ALT activities; (**G**) fasting glucose levels; (**H**) fasting insulin levels; (**I**) insulin resistance index; (**J**) insulin sensitivity index; (**K**) OGTT; and (**L**) AUC. The different lowercase letters (a, b, c, d) indicate significant differences between values in different groups (*p* < 0.05).

As outlined in Figure 4A–D, the serum TC, TG, and LDL-C levels in the HFD group were markedly increased, while the HDL-C levels were significantly decreased compared to the ND group (*p* < 0.05). Notably, the HEP intervention markedly reversed these trends, in which HEP dose-dependently reduced the serum TG and LDL-C levels. Unfortunately, the serum TG, TC, and LDL-C levels were markedly higher, and the HDL-C levels were lower in the HEP-treated group compared with the ND group (Figure 4A–D, *p* < 0.05). Additionally, liver injury in obese mice was also assessed by measuring serum ALT and AST activities. Compared with the ND group, serum ALT and AST activities in the HFD-fed mice were elevated by 1.63-fold and 2.12-fold (Figure 4E,F, *p* < 0.05). However, the HEP intervention remarkedly decreased ALT and AST activities (*p* < 0.05).

### 3.6. Effect of HEP on Insulin Resistance in Obese Mice

In comparison to the ND group, the serum glucose and insulin levels in the HFD group were elevated (Figure 4G,H, *p* < 0.05). However, both the serum glucose and insulin levels in the HEP treatment groups were remarkably reduced (*p* < 0.05). In comparison with the ND group, HOMA-IR was significantly elevated, and HOMA-IS was decreased in the HFD group, indicating that a chronic high-fat diet has led to insulin resistance in obese mice (Figure 4I,J, *p* < 0.05). Encouragingly, HEP significantly reduced the HOMA-IR and elevated the HOMA-IS in obese mice (Figure 4I,J, *p* < 0.05). Additionally, after glucose administration, the blood glucose levels in all groups rapidly increased within 30 min (Figure 4K, *p* < 0.05). Then, the blood glucose levels in the HFD group began to slowly decrease but remained high at 120 min. Conversely, the glucose levels in the HEP treatment groups declined faster than those in the HFD group (Figure 4K, *p* < 0.05). Additionally, compared with the ND group, the AUC in the HFD group was remarkably higher, whereas HEP dose-dependently decreased the AUC (Figure 4L, *p* < 0.05).

### 3.7. Effect of HEP on Hepatic Oxidative Stress in Obese Mice

**Figure 5:**
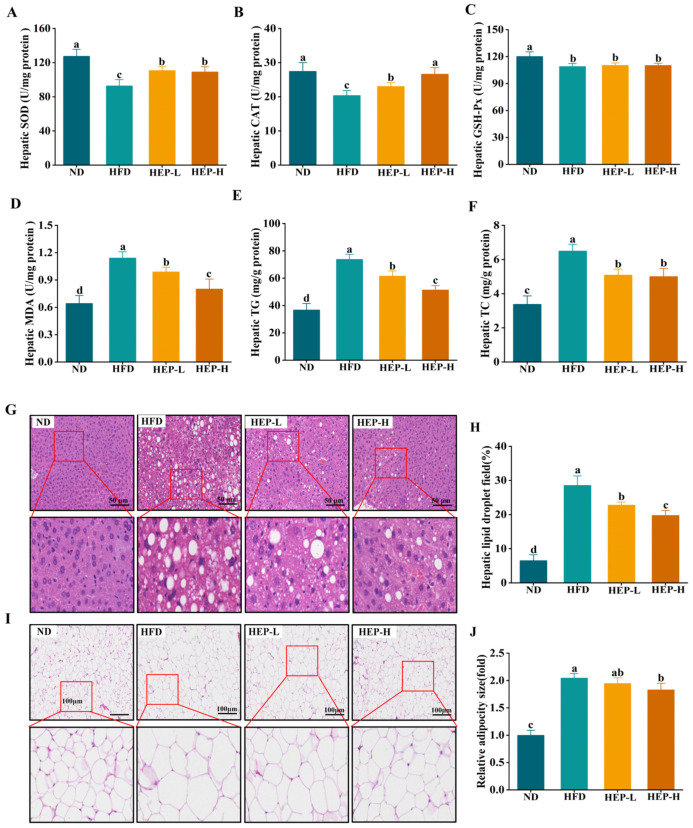
Effect of HEP on oxidative stress and lipid levels for liver and epididymal adipose in obese mice: (**A**) Hepatic SOD activities; (**B**) hepatic CAT activities; (**C**) hepatic GSH-Px levels; (**D**) hepatic MDA levels; (**E**) hepatic TG levels; (**F**) hepatic TC levels; (**G**) histopathology of the liver; (**H**) quantification of hepatic lipid droplet area; (**I**) histopathology of the epididymal adipose; and (**J**) relative adipocyte size of epididymal adipose. The different lowercase letters (a, b, c, d) indicate significant differences between values in different groups (*p* < 0.05).

SOD, CAT, and GSH-Px constitute the defense system against oxidative stress in organisms. As shown in Figure 5A–C, the levels of SOD, CAT, and GSH-Px in the HFD group were reduced by 27.3%, 25.9%, and 9.3% compared with the ND group, respectively (*p* < 0.05). The HEP intervention dose-dependently enhanced SOD and CAT activities in obese mice. However, the HEP intervention did not alter the hepatic GSH-Px levels compared to the HFD group (*p* > 0.05). Compared with the ND group, the MDA levels in the HFD group were elevated by 1.78-fold, while the HEP intervention dose-dependently reduced the MDA levels (Figure 5D, *p* < 0.05). Therefore, these results demonstrated that HEP could increase SOD and CAT activities, thereby mitigating HFD-induced hepatic oxidative stress injury.

### 3.8. Effect of HEP on Histopathology of Liver and Adipose Tissue

As exhibited in Figure 5E,F, the hepatic TG and TC levels in the HFD group were markedly increased compared to that of the ND group (*p* < 0.05). Encouragingly, the HEP intervention remarkably reduced the hepatic TG and TC levels in obese mice. However, the liver TG and TC levels in the HEP-treated groups did not reduce to normal levels (*p* < 0.05). As shown in Figure 5G, the hepatocytes in the normal mice exhibited normal structure, orderly arrangement, and obvious nuclei. In contrast, hepatocytes in the HFD group showed numerous vacuoles, and the lipid droplet area was increased by 4.41-fold compared with the ND group (Figure 5G,H, *p* < 0.05). The HEP intervention dose-dependently reduced the lipid droplet area of hepatocytes (Figure 5G,H, *p* < 0.05). In addition, the adipocyte morphology in mice is shown in Figure 5I. The adipocytes in obese mice were irregularly arranged, and the size significantly increased compared with the ND group (*p* < 0.05). Encouragingly, the size of the adipocytes in the HEP-H group was reduced by 10.5% (Figure 5I,J, *p* < 0.05).

### 3.9. Effect of HEP on the Expression of Lipid Metabolism-Related Genes

**Figure 6:**
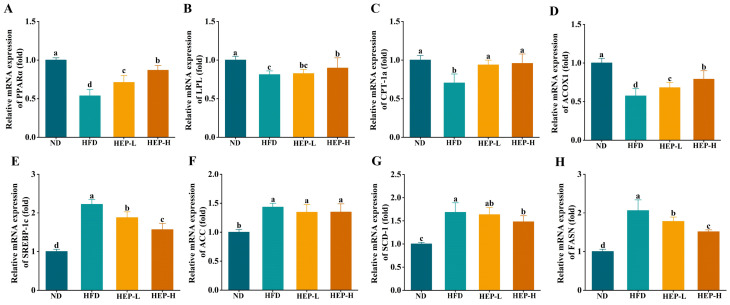
Effect of HEP on the mRNA expression of hepatic lipid metabolism-related genes in obese mice: (**A**) PPARα (fold); (**B**) LPL (fold); (**C**) CPT-1a (fold); (**D**) ACOX1 (fold); (**E**) SREBP-1c (fold); (**F**) ACC1 (fold); (**G**) SCD-1 (fold); and (**H**) FASN (fold). The different lowercase letters (a, b, c, d) indicate significant differences between values in different groups (*p* < 0.05).

To further investigate the underlying mechanism by which HEP ameliorated obesity, the mRNA expression of lipid metabolism-related genes in the liver was measured. Compared with the ND group, the mRNA expression of PPARα, LPL, CPT-1a, and ACOX1 in the HFD group was decreased by 46.3%, 18.8%, 29.7%, and 42.7% (Figure 6A–D, *p* < 0.05). Conversely, the mRNA expression of SREBP-1c, ACC1, SCD-1, and FASN in obese mice was upregulated by 2.22-fold, 1.68-fold, 1.43-fold, and 2.06-fold (Figure 6E–H, *p* < 0.05). In comparison with the HFD group, the HEP intervention significantly upregulated the mRNA expression of PPARα, LPL, CPT-1a, and ACOX1 and downregulated the mRNA expression of SREBP-1c, SCD-1, and FASN (*p* < 0.05). However, the mRNA expression of PPARα, LPL, ACOX1, SREBP-1c, SCD-1, and FASN in the HEP-treated groups did not reduce to normal levels (*p* < 0.05). Unfortunately, the HEP intervention did not alter the mRNA expression of acetyl-CoA carboxylase 1 (ACC1) (Figure 6F, *p* > 0.05).

### 3.10. Effect of HEP on the Expression of Lipid Metabolism-Related Protein

**Figure 7:**
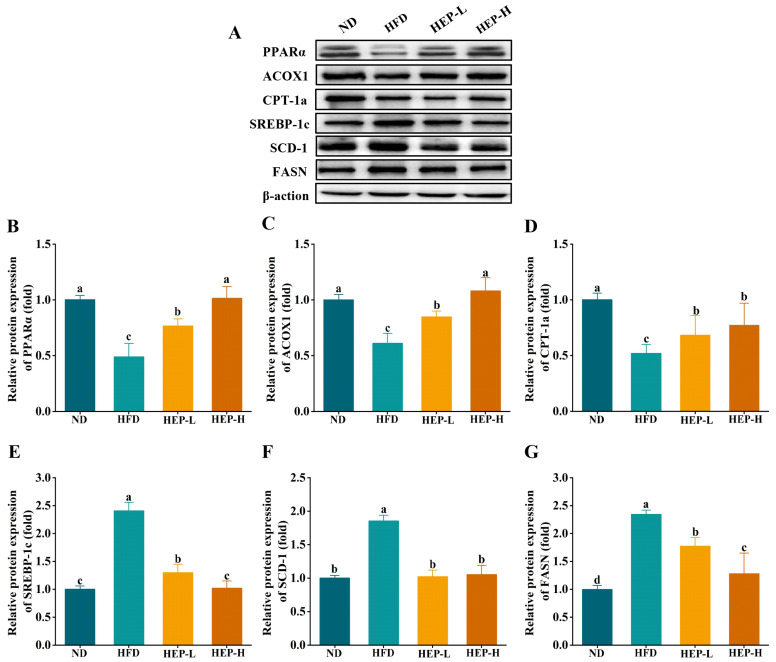
Effects of HEP on the protein expression of hepatic lipid metabolism-related genes in obese mice: (**A**) Representative images of Western blot; (**B**) PPARα (fold); (**C**) CPT-1a (fold); (**D**) ACOX1 (fold); (**E**) SREBP-1c (fold); (**F**) SCD-1 (fold); and (**G**) FASN (fold). The different lowercase letters (a, b, c, d) indicate significant differences between values in different groups (*p* < 0.05).

As outlined in Figure 7A–D, the protein expression of PPARα, CPT-1a, and ACOX1 in the HFD group was downregulated by 51.2%, 48.2%, and 39.0% compared with the ND group, while the HEP intervention reversed these trends. Notably, the PPARα and ACOX1 protein levels in the HEP-H group were restored to normal levels (Figure 7A–C, *p* > 0.05). Additionally, the protein expression of SREBP-1c, SCD-1, and FASN in the HFD group was remarkably upregulated by 2.4-fold, 1.85-fold, and 2.35-fold (Figure 7A,E–G, *p* < 0.05). The HEP intervention significantly downregulated the protein expression of SREBP-1c, SCD-1, and FASN in obese mice (Figure 7A,E–G, *p* < 0.05). Notably, the SREBP-1c and SCD-1 protein levels in the HEP-H group were restored to normal levels (Figure 7E,F, *p* > 0.05). Thus, these results suggested that HEP could upregulate the protein expression of PPARα, CPT-1a, and ACOX1 and downregulate the protein expression of SREBP-1c, SCD-1, and FASN, thereby alleviating obesity.

## 4. Discussion

Obesity poses a serious threat to individual health and is a major challenge to global public health. While traditional chemically synthesized drugs can achieve short-term weight reduction, their severe side effects limit their feasibility for long-term use. Consequently, exploring natural and safer alternatives has become a focal point in obesity treatment. *Hericium erinaceus*, a nutritious medicinal and edible mushroom, contains various bioactive components and exhibits notable health-promoting effects. Although *Hericium erinaceus* is rich in protein, the anti-obesity mechanisms of its proteins, compared to its polysaccharides and dietary fibers, remain largely unexplored. Therefore, this study aims to investigate the effect and potential mechanism of *Hericium erinaceus* protein (HEP) in combating obesity.

Obesity primarily results from a prolonged imbalance between energy ingestion and energy consumption. A long-term HFD disrupts glucose and lipid metabolism in multiple organs, leading to a fatty liver and a substantial accumulation of subcutaneous and visceral fat [25]. In this study, 8 weeks of HFD treatment resulted in an abnormal weight gain in mice (Figure 2). As expected, the HEP intervention markedly reduced body weight gain in obese mice (Figure 1). Furthermore, the organ weight is positively correlated with obesity [24]. Interestingly, despite HEP reducing the body weight and organ indexes, HEP did not alter the appetite (Figure 1). Previous studies indicated that α-lactalbumin prevented abnormal increases in body weight and the organ index in HFD-fed mice without affecting their food intake, which aligns with our finding [3]. Therefore, our findings indicated that HEP mitigated HFD-induced obesity without altering the appetite.

Dietary fats with high caloric content are the primary energy source for the organism. Intestinal lipases play a key role in the digestion and absorption of these fats, with pancreatic lipase being the key enzyme responsible for hydrolyzing 50–70% of the dietary fats in the intestinal lumen [26]. After excessive fat intake by the organism, pancreatic lipase hydrolyses dietary fats into monoacylglycerol and free fatty acids (FFAs), which are subsequently absorbed in the intestines and then re-synthesized into new fats, leading to ectopic accumulation of fats in the organs and ultimately causing obesity [27]. Therefore, inhibiting pancreatic lipase activity could aid in weight loss. In this study, pancreatic lipase activity in the HFD group was increased, indicating higher fat hydrolysis efficiency in obese mice (Figure 3). Conversely, pancreatic lipase activity in the HEP intervention groups was significantly lower, while fecal TG levels were significantly higher, suggesting that HEP reduced dietary fat absorption by inhibiting pancreatic lipase activity (Figure 3). A previous study showed that Lactobacillus plantarum HF02 decreased pancreatic lipase activity, thereby enhancing TG excretion and reducing the liver and serum lipid levels in obese mice, which is consistent with our findings [24]. Consistently, peptides obtained from simulated digestion of adzuki bean protein have demonstrated excellent pancreatic lipase inhibitory activity [26]. Therefore, the pancreatic lipase inhibitory peptide released from HEP in the gut may be one of its potential mechanisms against obesity. In addition, a previous study indicated that α-Lactalbumin peptides reduced the ratio of Firmicutes to Bacteroidetes and subsequently reduced energy intake in high-fat diet-fed mice, thereby alleviating insulin resistance and dyslipidemia, which is consistent with our results [28]. Hence, the underlying mechanism needs to be further investigated in the future.

Liver lipid metabolism is one of the most vital factors in maintaining normal lipid metabolism in the body. Enhancing liver fatty acid oxidation and suppressing lipid synthesis are considered effective strategies for alleviating obesity [25]. PPARα is a vital regulator of lipid metabolism and has been identified as an important therapeutic target for obesity. It has been reported that PPARα activation upregulates its target genes (LPL, ACOX1, and CPT-1a) expression, enhancing lipid degradation and fatty acid oxidation, which ameliorates hepatic steatosis, dyslipidemia, and insulin resistance [29]. Among these, LPL catalyzes the degradation of TG into glycerol and FFAs [30]. ACOX1 is the rate-limiting enzyme of peroxisomal fatty acid oxidation, initiating the oxidation of long-chain fatty acids [3]. CPT-1a facilitates the transport of long-chain fatty acids into mitochondria, accelerating mitochondrial fatty acid oxidation [31]. In the present study, chronic HFD feeding suppressed PPARα activation in the liver, which, in turn, downregulated lipolysis and fatty acid oxidation-related gene levels (LPL, CPT-1a, and ACOX1) (Figure 6 and Figure 7) and ultimately led to hepatic lipid deposition and dyslipidemia (Figure 4 and Figure 5). As expected, the HEP intervention significantly upregulated the mRNA and protein expression of PPARα in the liver, subsequently increasing the mRNA and protein expression of LPL, ACOX1, and CPT-1a (Figure 6 and Figure 7), thereby decreasing the serum and liver lipid levels (Figure 4 and Figure 5). The histopathological findings further supported that HEP attenuated lipid deposition in the liver and adipose tissue (Figure 5). Ethanolic extracts from *Hericium erinaceum* could reduce the serum TC, LDL-C, and TG levels and elevate the HDL-C levels in hyperlipidemic rats, which is consistent with our findings [13]. Consistently, Chen et al. demonstrated that α-lactalbumin peptide DQW activated PPARα and increased the expression of ACOX1 and CPT-1a, thereby accelerating fatty acid oxidation in lipid-accumulating HepG2 cells [32]. Therefore, our results suggested that HEP promoted fatty acid oxidation in the liver of obese mice by activating PPARα.

On the other hand, PPARα can also regulate hepatic lipogenesis pathways by modulating the expression of SREBP-1c [33]. SREBP-1c is a vital transcriptional regulator of fatty acids and cholesterol synthesis. When activated, it upregulates the expression of critical fatty acid synthesis genes (ACC1, SCD-1, and FASN), thereby accelerating de novo lipid synthesis [25]. ACC1 catalyzes the carboxylation of acetyl-CoA to malonyl-CoA, which is utilized in lipid synthesis [3]. SCD-1 is involved in the synthesis of palmitate from acetyl-CoA and forms double bonds in stearoyl-CoA, thus facilitating the formation of long-chain fatty acids [34]. FASN catalyzes the final step of fatty acid biosynthesis, using malonyl-CoA to produce saturated fatty acid palmitate, which is subsequently used to synthesize triglycerides [32]. In this study, the expression levels of SREBP-1c, ACC1, SCD-1, and FASN were significantly upregulated in HFD-fed mice (Figure 6 and Figure 7). Therefore, HFD feeding accelerated the hepatic lipid synthesis process in obese mice, resulting in liver lipid deposition and dyslipidemia (Figure 4 and Figure 5). Encouragingly, HEP downregulated the SREBP-1c, FASN, and SCD-1 expression levels (Figure 6 and Figure 7). Meanwhile, the liver and serum lipid level reductions further supported the idea that HEP attenuated adipogenesis in obese mice (Figure 4 and Figure 5). Consistently, a previous study demonstrated that ursolic acid decreased SREBP-1c, FASN, and ACC1 expression levels via activating PPARα in the liver, alleviating liver lipid accumulation and dyslipidemia in obese rats [35]. Proanthocyanidin extracted from Chinese bayberry leaves (BLPs) activated amp-activated protein kinase (AMPK) and ameliorated gut flora disruption, downregulated adipogenesis-related gene expression and upregulated β-oxidation-related gene expression, thereby reducing obesity in HFD-fed mice [36]. Conformably, Gong et al. indicated that Compound Oolong tea improved hepatic lipid metabolism by activating the AMPK-PPAR pathway and regulating the gut microbiota dysbiosis, thereby attenuating dyslipidemia in obese mice [37]. *Hericium erinaceus* insoluble dietary fiber increased the abundance of *Akkermansia* and *norank_f_Muribaculaceae* in the gut microbiota of obese mice, subsequently decreasing the production of metabolites such as triglycerides, bile acids and their derivatives, thereby ameliorating obesity [14]. Thus, the gut microbiota plays an important role in anti-obesity therapy. There is a necessity for future studies to investigate whether HEP exerts beneficial effects on obesity by modulating the gut microbiota.

Insulin resistance, commonly associated with obesity, is a precursor to cardiovascular diseases, type II diabetes, and metabolic disorders [38]. A long-term HFD leads to excessive lipid influx into the liver and over-secretion of pro-inflammatory factors, thereby disrupting the insulin signaling pathway and enhancing hepatic gluconeogenesis, resulting in hyperglycemia [28]. In turn, insulin resistance elevates the serum FFA levels, increasing hepatic FFA influx and stimulating lipid accumulation and cholesterol synthesis, ultimately causing dyslipidemia and creating a vicious cycle [3]. In this study, obese mice exhibited significantly increased fasting blood glucose and insulin levels and a HOMA-IR index, while the HOMA-IS index and glucose tolerance were significantly reduced, indicating a state of insulin resistance (Figure 5). As expected, the HEP intervention significantly reversed these trends (Figure 4). Previous studies have demonstrated that long-term dietary intervention with fenofibrate (a PPARα agonist) reduced HFD-induced dyslipidemia and insulin resistance [39]. Moreover, PPARα activation improves hepatic lipid metabolism in HFD-fed mice, alleviating hepatic lipid deposition and insulin resistance [3]. Therefore, we hypothesized that HEP might mitigate liver lipid deposition via activating PPARα, thereby alleviating insulin resistance.

Excessive influx of FFAs into the liver can lead to lipid accumulation in hepatocytes, stimulating the production of reactive oxygen species (ROS) and leading to oxidative stress [40]. Mitochondria, the primary organelles responsible for lipid metabolism, suffer functional impairment due to oxidative stress, which inhibits mitochondrial fatty acid oxidation, thereby disrupting lipid homeostasis [25]. SOD, CAT, and MDA are key markers in the evaluation of oxidative stress. MDA is the final product of lipid peroxidation, while SOD and CAT are key enzymes that convert ROS into water and oxygen [25]. In this study, HEP exhibited antioxidant capacity in vitro, suggesting its potential to mitigate oxidative stress injury (Figure 2). Consistent with the in vitro antioxidant results, the HEP intervention significantly increased hepatic SOD and CAT activities in obese mice, subsequently reducing MDA levels (Figure 5). Additionally, the decreased serum ALT and AST activities further supported the idea that HEP could attenuate liver damage (Figure 4). A previous study indicated that *Hericium erinaceus* peptide KSPLY increased CAT, SOD, and GSH-Px activities, thereby alleviating H_2_O_2_-induced oxidative stress in HepG2 cells, which is consistent with our study [41]. These findings indicated that HEP could attenuate oxidative stress injury in the liver by modulating the antioxidant defense system. Notably, a previous study showed that PPARα activation could upregulate hepatic SOD and CAT expression, enhancing antioxidant capacity and playing a crucial role in protecting hepatocytes from injury [42]. Consistently, Fidaleo et al. reported that cocoa ameliorated hepatic fatty acid oxidation and enhanced SOD and CAT activities in obese mice via activating the PPARα pathway, reducing ectopic lipid accumulation and oxidative stress injury [43]. Therefore, our results suggested that HEP protected hepatocytes from HFD-induced oxidative stress injury by activating the PPARα pathway.

## 5. Conclusions

**Figure 8:**
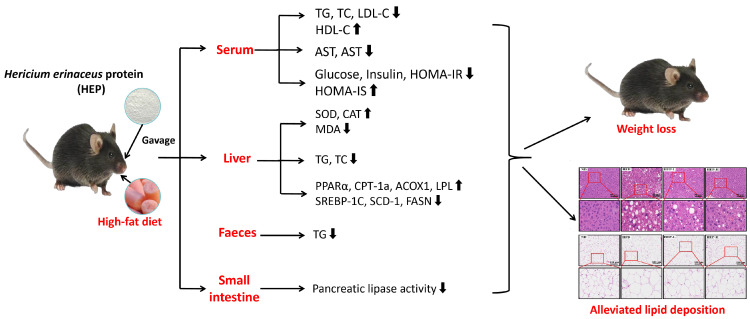
The underlying mechanism by which HEP attenuates HFD-induced body weight gain and lipid metabolism disorders in obese mice (↑: upregulation, ↓: downregulation).

In summary, this study is the first to investigate the protective effects of HEP against HFD-induced obesity. In vitro, HEP demonstrated significant antioxidant activity. In vivo, the HEP treatment alleviated HFD-induced body weight gain, hepatic steatosis, dyslipidemia, and oxidative stress injury in obese mice. This effect may be attributed to two mechanisms: firstly, the HEP treatment inhibited intestinal lipase activity, reducing the dietary fat absorption of obese mice; secondly, the HEP treatment activated the PPARα signaling pathway, improving hepatic lipid metabolism-related mRNA and protein expression, thereby restoring lipid metabolism homeostasis (Figure 8). It is noteworthy that after gastrointestinal digestion, HEP is absorbed in the form of amino acids and bioactive peptides. Additionally, although our results indicated that HEP could alleviate dyslipidemia and lipid deposition in obese mice, animal models of obesity do not fully reflect human obesity [25]. Therefore, further clinical and bioavailability studies will be necessary to confirm the clinical safety and side effects of HEP, as well as its underlying mechanisms for alleviating obesity. In conclusion, this study provides a theoretical basis for the application of HEP in the functional food industry.

## Data Availability

The original contributions presented in the study are included in the article/Supplementary Material, further inquiries can be directed to the corresponding author.

## Supplementary Materials

The following supporting information can be downloaded at: https://www.mdpi.com/article/10.3390/foods14030459/s1, Table S1: The detailed diet ingredients of the mice diet; Table S2: Details of the sequences of the primers for this study; Table S3: The components of HEP.
