# Supplementary material for: Hericium erinaceus Protein Alleviates High-Fat Diet-Induced Hepatic Lipid Accumulation and Oxidative Stress In Vivo
Source: Foods. 2025 Jan 31;14(3):459. doi: 10.3390/foods14030459 (PMC11817179; doi:10.3390/foods14030459)
Supplement: Supplementary file 1 [file foods-14-00459-s001.zip › foods-3391626-supplementary.pdf]

Supporting material

Supplementary Table. S1 The detailed diet ingredients of the mice diet.

Ingredient	Normal diet (gm%)	High-fat diet (gm%)
Casein	200	200
L-Cystine	3	3
Sucrose	350	68.8
Maltodextrin	35	125
Corn Starch	351	0
Cellulose	50	50
Soybean oil	25	25
Lard	20	245
Potassium citrate	16.5	16.5
Dicalcium phosphate	13	13
Calcium carbonate	5.5	5.5
Vitamin Mix	10	10
Choline bitartrate	2	2
Mineral Mix	10	10
Total (kcal/g)	3.85	5.24

Supplementary Table. S2 Details of the sequences of the primers for this study.

Gene	Forward primer (5’ - 3’)	Reverse primer (5’ - 3’)
PPARα	F: AGGCTGTAAGGGCTTCTTTC	R: GCATTTGTTCCGGTTCTTCTTC
CPT-1a	F: AGATCAATCGGACCCTAGACAC	R: CAGCGAGTAGCGCATAGTCA
ACOX1	F: CGCACATCTTGGATGGTAGT	R: GGCTTCGAGTGAGGAAGTTATAG
LPL	F: CCCTACAAAGTGTTCCATTACCAA	R: TTGTGTTGCTTGCCATCCTCA
SREBP-1c	F: GCAGCCACCATCTAGCCTG	R: CAGCAGTGAGTCTGCCTTGAT
ACC1	F: GTTTCTCTGGTGGGATGAAAGA	R: GGGCGGGATGTAAACCATTA
SCD-1	F: TTCTTGCGATACACTCTGGTGC	R: CGGGATTGAATGTTCTTGTCGT
FASN	F: AGACCCGAACTCCAAGTTATTC	R: GCAGCTCCTTGTATACTTCTCC
β-actin	F: GGCTGTATTCCCCTCCATCG	R: CCAGTTGGTAACAATGCCATGT

Supplementary Table. S3 The components of HEP

Parameters	LSP
Protein content (%)	86.57 ± 0.67
Water (%)	6.03 ± 0.21
Carbohydrate (%)	4.67 ± 0.34
Ash (%)	2.28 ± 0.06
